# Brain circuits for promoting homeostatic and non-homeostatic appetites

**DOI:** 10.1038/s12276-022-00758-4

**Published:** 2022-04-26

**Authors:** Benjamin Hyunju Ahn, Minyoo Kim, Sung-Yon Kim

**Affiliations:** 1 Department of Chemistry, Seoul National University, Seoul, 08826 South Korea; 2 Institute of Molecular Biology and Genetics, Seoul National University, Seoul, 08826 South Korea

**Keywords:** Feeding behaviour, Reward, Feeding behaviour, Obesity

## Abstract

As the principal means of acquiring nutrients, feeding behavior is indispensable to the survival and well-being of animals. In response to energy or nutrient deficits, animals seek and consume food to maintain energy homeostasis. On the other hand, even when animals are calorically replete, non-homeostatic factors, such as the sight, smell, and taste of palatable food, or environmental cues that predict food, can stimulate feeding behavior. These homeostatic and non-homeostatic factors have traditionally been investigated separately, but a growing body of literature highlights that these factors work synergistically to promote feeding behavior. Furthermore, recent breakthroughs in cell type-specific and circuit-specific labeling, recording, and manipulation techniques have markedly accelerated the discovery of well-defined neural populations underlying homeostatic and non-homeostatic appetite control, as well as overlapping circuits that contribute to both types of appetite. This review aims to provide an update on our understanding of the neural circuit mechanisms for promoting homeostatic and non-homeostatic appetites, focusing on the function of recently identified, genetically defined cell types.

## Appetite: Linking neural circuits to feeding behaviors

Research on the neural circuit mechanisms underlying feeding behaviors is critical to identifying therapeutic targets for food-related disorders like obesity and anorexia. Sung-Yon Kim and colleagues at Seoul National University, South Korea, reviewed the current understanding of neural circuits promoting feeding behavior, which is regulated by homeostatic and non-homeostatic appetites. In response to deficits in energy (caloric) or nutrients, specific populations of neurons sensitive to hormones leptin and ghrelin generate homeostatic appetite and promote feeding. In addition, diverse neural populations stimulate non-homeostatic appetite in the absence of immediate internal needs and are thought to drive overconsumption in the modern obesogenic environment. These appetites extensively interact through overlapping neural circuits to jointly promote feeding behaviors.

## Introduction

Food intake is essential to the survival of animals as a critical contributor to energy homeostasis^1,2^. While self-evident in our daily lives, the importance of this vital function is further emphasized by the fact that inappropriate regulation of feeding behavior is a hallmark of serious metabolic and psychiatric conditions, such as obesity and anorexia^3–5^. As such, decades of research have delved into the neural circuit mechanisms for appetite—the motivation to eat.

In response to caloric deficiency, animals seek and consume food to defend energy homeostasis^1,2,6–9^. Information on energy stores and nutrient availability is thought to be primarily conveyed by peripherally produced hormones to certain central neurons^1,2,4,7–9^. These interoceptive hormones can directly reach central neurons behind the blood–brain barrier (BBB) via several mechanisms within circumventricular organs (CVOs; e.g., the median eminence in the hypothalamus and area postrema in the hindbrain) that permit passive transport of hormones to these neurons^2,10^. In addition, these hormones can indirectly signal the energy state to hindbrain neurons by acting on vagal afferent neurons^2,5,8^. Upon direct and indirect exposure to peripheral signals of energy or nutrient deficit, central neurons that support homeostatic appetite are activated and drive feeding^1,2,11^.

Feeding behavior can also be initiated in the absence of apparent homeostatic needs^1,2,9,11–13^. For example, the sight, smell, and taste of palatable foods, as well as contextual cues that predict food reward, can all stimulate appetite even when animals are calorically replete^1,2,11,12^. Diverse neural populations mediating this non-homeostatic appetite have been identified and characterized thus far^1,2,12,13^. Some of these neurons drive feeding behavior specific to palatable foods regardless of the internal state^2,4,9,11–13^, while some populations are shown to promote food intake in response to conditioned sensory cues even in a sated state^12,13^. This mode of appetite is thought to encourage overconsumption beyond current physiological needs, possibly as a preventive mechanism against anticipated energy deficit^1,2,12^. However, in the modern obesogenic environment where we face an abundance, rather than a scarcity of food, this non-homeostatic appetite is considered to underlie the etiology of obesity, highlighting its clinical relevance^4,11^.

Much research has described these different appetites in isolation, with the predominant assumption that homeostatic and non-homeostatic appetites are mutually exclusive entities^1,6^. However, it has continuously been noted that these appetites frequently interact with each other to promote feeding behavior; for one example, energy deficits increase the incentive salience of food-predictive cues^1,4,9,11,13^. Accordingly, recent studies have found that the activity of neural populations that are well established to promote non-homeostatic appetite is also modulated by the hunger state and that the neurons essential to homeostatic appetite also respond to non-homeostatic factors^1,11,14^. In addition, many neural populations that play a pivotal role in both homeostatic and non-homeostatic appetite regulation have been identified^9,11^.

Recent advancements in techniques enabling anatomical and functional interrogation of genetically defined neuron types have markedly facilitated the disentanglement of well-defined neural circuit components mediating the control of homeostatic and non-homeostatic appetites^14–16^. Here, we review the body of knowledge on the neural mechanisms promoting feeding, focusing on the latest progress under the following three sections: (1) Brain circuits for homeostatic appetite, (2) Brain circuits for non-homeostatic appetite, and (3) Interactions between homeostatic and non-homeostatic appetites. We also discuss future directions that would provide further insights into a comprehensive understanding of the neural mechanisms underlying the generation of appetite and feeding behavior.

## Brain circuits for homeostatic appetite

Several hormones produced from peripheral metabolic organs primarily transmit information on energy stores and nutrient availability^8^. A representative example is leptin, which is an anorexigenic hormone released by adipocytes into the blood circulation^17^. Plasma levels of leptin largely correlate with the body’s fat stores^17^. Systemic administration of leptin decreases food intake and induces weight loss, whereas in animals lacking the leptin gene, food consumption and body mass are dramatically increased, and this phenotype is fully reversed by leptin treatment^17^. Another example is ghrelin, which is an orexigenic hormone secreted by enteroendocrine cells in the stomach^1^. The levels of ghrelin peak before a meal and drop to baseline levels in less than an hour after eating^18^. The levels of plasma ghrelin increase following prolonged caloric restriction, whereas the extent to which ghrelin levels drop after a meal correlates with caloric load^18^. Curiously, while ghrelin administration sufficiently increases food intake, ghrelin-deficient mice exhibit no significant differences in standard chow intake^18^, perhaps due to other orexigenic mechanisms that prevent malnutrition in animals.

**Fig. 1:**
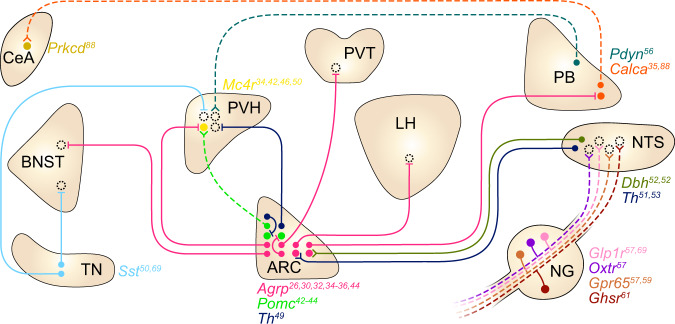
Summary of the circuits for homeostatic appetite. Each circle denotes a neural population that sends or receives signals involved in homeostatic appetite. Filled circles include genetically defined populations, whereas broken circles indicate projection targets of unknown identity. Solid and dashed lines indicate connections that are orexigenic and anorexigenic when artificially activated, respectively. Split terminals indicate activation of target neurons, and bar terminals indicate inhibition of target neurons. Specific marker genes are indicated by color-coded labels adjacent to regions that contain soma of each population. Note that not all connections depicted here have been confirmed to be monosynaptic. The listed genes encode *Agrp*, agouti-related peptide; *Pomc*, pro-opiomelanocortin; *Pdyn*, prodynorphin; *Calca*, calcitonin/calcitonin-related polypeptide, alpha; *Th*, tyrosine hydroxylase; *Mc4r*, melanocortin 4 receptor; *Glp1r*, glucagon-like peptide 1 receptor; *Dbh*, dopamine β-hydroxylase; *Ghsr*, growth hormone secretagogue receptor; *Drd1*, dopamine receptor D1; *Sst*, somatostatin; *Oxtr*, oxytocin receptor; *Gpr65*, G-protein-coupled receptor 65; *Prkcd*, protein kinase C delta. Abbreviations: BNST, bed nucleus of the stria terminalis; CeA, central amygdaloid nucleus; TN, tuberal nucleus of the hypothalamus; PVH, paraventricular hypothalamic nucleus; ARC, arcuate hypothalamic nucleus; PVT, paraventricular thalamic nucleus; LH, lateral hypothalamic area; NG, nodose ganglion; PB, parabrachial nucleus; NTS, nucleus of the solitary tract.

Central neurons that respond to these hormones have been found proximal to or within CVOs^10^. Most prominently, neurons expressing the gene encoding agouti-related peptide (AgRP; encoded by the *Agrp* gene) in the arcuate nucleus (ARC^*Agrp*^ neurons), which reside near the BBB-lacking median eminence, detect changes in both leptin and ghrelin levels^17–19^ (Fig. 1). Increases in leptin lead to decreased activity of ARC^*Agrp*^ neurons in leptin-deficient and leptin haplo-insufficient mice^20^, and application of leptin ex vivo decreases the firing rate of ARC^*Agrp*^ neurons from fasted wild-type mice^21^. Conversely, ghrelin potently increases the activity of ARC^*Agrp*^ neurons both in vivo and ex vivo^22,23^. Consistent with their responses to ghrelin and leptin, the overall activity of ARC^*Agrp*^ neurons encodes the hunger state, as these neurons are highly active in the fasted state but inactive during the fed state^22,24^.

ARC^*Agrp*^ neurons are well established to causally drive homeostatic appetite. These neurons are necessary for the intake of nonpalatable chow but dispensable for palatability-driven feeding^25–29^; specific ablation of ARC^*Agrp*^ neurons leads to starvation when animals are fed standard chow^25–27^, but this does not significantly affect the intake of a palatable high-fat, high-sugar diet^28,29^. Artificial activation of ARC^*Agrp*^ neurons reliably evokes feeding behavior^30,31^, likely by recruiting noncollateralizing inhibitory projections to the paraventricular hypothalamus (PVH), lateral hypothalamus (LH), bed nucleus of the stria terminalis (BNST), and paraventricular thalamus (PVT)^32–36^, but which of these projections are required for the orexigenic effect of ARC^*Agrp*^ neuron stimulation remains to be determined. Furthermore, ARC^*Agrp*^ neurons are critical for the orexigenic effect of ghrelin and the anorexigenic effect of leptin. Genetic ablation of leptin receptor (LepR) in ARC^*Agrp*^ neurons via the CRISPR–Cas9 system increases food intake and body weight to a similar extent as mice lacking LepR completely^37^. These striking findings imply that leptin predominantly acts on ARC^*Agrp*^ neurons—among its many potential targets—to maintain energy balance^37^. In addition, ARC^*Agrp*^ neurons at least partially mediate ghrelin-evoked feeding. Ablating ARC^*Agrp*^ neurons abrogates the increase in feeding that follows both oral administration of a ghrelin receptor agonist and subcutaneous injection of ghrelin^29,38^. In mice lacking *Ghsr*, the gene encoding growth hormone secretagogue receptor (GHSR), however, reinstating *Ghsr* specifically in ARC^*Agrp*^ neurons only partially restores the orexigenic effects of subcutaneously injected ghrelin^39^. These results collectively suggest that ARC^*Agrp*^ neurons are required but not sufficient for ghrelin-evoked feeding.

ARC neurons expressing the pro-opiomelanocortin-encoding gene (*Pomc*) (ARC^*Pomc*^ neurons) are another key population that responds to hormones signaling the energy state, and that plays an important role in homeostatic appetite^40–42^. In contrast to ARC^*Agrp*^ neurons, the activity of ARC^*Pomc*^ neurons decreases upon fasting and ghrelin injection and increases upon food intake and leptin treatment^22,41^, although recent single-cell RNA sequencing and electrophysiological experiments have revealed substantial heterogeneity in the responses of ARC^*Pomc*^ neurons to leptin^43^. This antagonistic activity of ARC^*Pomc*^ and ARC^*Agrp*^ neurons appears to be shaped by a mechanism analogous to the flip-flop model from digital electronics^44^. When mice are calorically replete, ARC^*Pomc*^ neurons are activated, which in turn decreases the activity of glutamatergic terminals presynaptic to ARC^*Agrp*^ neurons by releasing β-endorphin, leading to reduced ARC^*Agrp*^ neuronal activity (although the identity of the upstream glutamatergic neurons remains to be studied)^44^. Conversely, when mice are energy deficient, an increase in ghrelin levels activates *Ghsr*-expressing upstream glutamatergic terminals by activating an intracellular positive feedback loop involving AMP-activated protein kinase^44^. When the intracellular loop is initiated, ARC^*Agrp*^ neurons are activated and maintain their elevated activity even after ghrelin is washed out, which inhibits ARC^*Pomc*^ neurons by the release of GABA^44^. This elevated activity remains persistent until ARC^*Pomc*^ neurons are activated—one suggested mechanism is via leptin, although this might not be physiologically relevant given that the leptin level does not acutely increase after food intake. This interesting study illustrates a neural mechanism by which ARC^*Pomc*^ and ARC^*Agrp*^ neurons maintain their sustained activity in a bistable manner that is flipped by an energy excess and deficit, represented by leptin and ghrelin^44^.

In addition to their activity dynamics, the effects of ARC^*Pomc*^ neurons on appetite, as well as the effects of their neuropeptides on melanocortin receptors expressed in downstream neurons, also oppose those of ARC^*Agrp*^ neurons^1,30,42,45,46^. Activation of ARC^*Pomc*^ neurons suppresses feeding^30,45^, whereas ablation of ARC^*Pomc*^ neurons induces massive obesity in mice^45^. α-Melanocyte stimulating hormone (α-MSH), a peptide product of the *Pomc* gene, acts as an agonist of melanocortin 3 and 4 receptors (MC3R and MC4R; melanocortin receptors known to be expressed in the brain^47^), whereas agouti-related peptide (AgRP) acts as an inverse agonist of these receptors^47^. Key convergent sites for these neurons are *Mc4r*-expressing neurons in the PVH (PVH^*Mc4r*^ neurons), of which the expression of MC4R is both necessary and sufficient for the control of feeding^48^. Application of α-MSH and AgRP to PVH^*Mc4r*^ neurons ex vivo increases and reduces their firing activity, respectively^46^.

Within or proximal to the ARC, several other neural populations are found to be orexigenic and activated by hunger and ghrelin^49,50^. One example is the neurons marked by the expression of tyrosine hydroxylase (encoded by the *Th* gene) in the ARC, which are distinct from ARC^*Agrp*^ and ARC^*Pomc*^ neurons. Optogenetic stimulation of this population robustly increases food intake, whereas tetanus toxin-mediated silencing of these neurons induces body weight loss^49^. In addition, these neurons show increased firing rates in the fasted state, as well as in response to ex vivo application of ghrelin^49^. These neurons inhibit ARC^*Pomc*^ neurons and neurons in the PVH, likely through the corelease of dopamine (DA) and GABA^49^; the genetic identity of PVH neurons postsynaptic to ARC^*Th*^ neurons remains to be determined.

As another example, in the subregion of the hypothalamus residing lateral to the ARC called the tuberal nucleus, inhibitory neurons expressing the somatostatin gene (*Sst*) (TN^*Sst*^ neurons) are activated by ghrelin both ex vivo and in vivo^50^. When activated, these neurons promote food intake, as chemogenetic activation of this population dramatically stimulates food consumption (by increasing both the meal duration and frequency). In contrast, chemogenetic and optogenetic inhibition of these neurons reduces food consumption (only by decreasing meal frequency), and ablation of these neurons attenuates body weight gain over ten weeks compared with controls. Optogenetic stimulation of the projection from the TN^*Sst*^ neurons either to the BNST or PVH was sufficient to stimulate appetite^50^.

In addition to these key neural populations in the hypothalamus, catecholaminergic neurons in the nucleus of the solitary tract (NTS) that express dopamine β-hydroxylase or tyrosine hydroxylase (encoded by the *Dbh* or *Th* gene) have been implicated in promoting homeostatic appetite. These neurons are activated following food deprivation^51^, and the level of *Dbh* mRNA in the NTS is increased upon peripheral ghrelin injection^52^. Optogenetic and chemogenetic activation of *Dbh*-expressing NTS neurons potently increases food intake, while optogenetic inhibition of these neurons suppresses feeding^51^. Some of the *Th*-expressing NTS neurons project to the ARC, and activation of this projection stimulates appetite while inhibiting this pathway impairs feeding elicited by low blood glucose levels^53^. Moreover, ablating ARC-projecting, *Dbh*-expressing neurons throughout the brain, which is also found to ablate most of the catecholaminergic neurons in the NTS, abolishes feeding evoked by peripheral injection of ghrelin^52^. The appetite-promoting effect of ARC-projecting NTS catecholaminergic neuron stimulation is mediated by norepinephrinergic transmission in the ARC, as this effect is abolished by pharmacological blockade of α-adrenergic receptors^53^ (although NPY may also contribute to this orexigenic effect, as deletion of the gene encoding NPY specifically in catecholaminergic NTS neurons also attenuated the increase in food intake evoked by stimulation of these neurons^51^). Together with the observation that bath application of norepinephrine activates ARC^*Agrp*^ neurons^53,54^, these results suggest that NTS catecholaminergic neurons induce feeding by recruiting ARC^*Agrp*^ neurons. Given that ARC^*Pomc*^ neurons are also inhibited by norepinephrine in the presence of synaptic blockers^54^, it is likely that norepinephrine release in the ARC elicits a strong orexigenic effect via concurrent activation of ARC^*Agrp*^ neurons and inhibition of ARC^*Pomc*^ neurons^54^.

Ghrelin also acts on vagal sensory neurons, many of which carry information about the nutrient composition of a meal or the mechanical distension of the gastrointestinal tract to central hindbrain neurons in the NTS^1,55–60^. Vagal sensory neurons are necessary for ghrelin-evoked food intake, as severing the vagal afferents that innervate visceral organs abolished the increase in food intake that follows systemic ghrelin administration^61^. Consistently, knockdown of *Ghsr* specifically in vagal sensory neurons blunted the response of these neurons to peripheral ghrelin^61^, indicating that ghrelin sensing in vagal sensory neurons requires the expression of GHSR.

Recent investigations have also identified specific circuits in the hypothalamus that drive nutrient-specific appetites in response to internal signals indicating the deficit of certain nutrients—thereby contributing to the maintenance of the levels of individual nutrients, in addition to the mechanisms for defending the homeostasis of the overall energy level^62^. For example, when animals are maintained on a high-carbohydrate and low-protein diet, the liver releases high levels of fibroblast growth factor 21 (FGF21) into the bloodstream^63,64^. Systemic administration of FGF21 decreases the preference for sugar against water^65^, and in mice presented with a choice of diets differing in protein composition, intracerebroventricular (i.c.v.) injection of FGF21 increases the preference for diets with a higher protein content^66^. Critically, the reduced carbohydrate preference upon increased FGF21 levels requires the expression of FGF21’s cognate receptor β-klotho in the brain^66^, specifically within the neurons in the ventromedial hypothalamus (expressing the region-specific marker *Sf1*), a region adjacent to the third ventricle and the ARC^67^.

## Brain circuits for non-homeostatic appetite

**Fig. 2:**
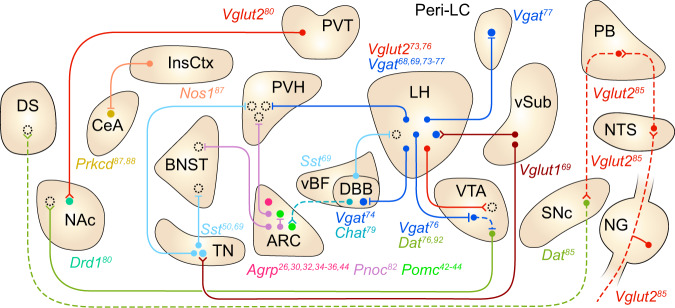
Summary of the circuits for non-homeostatic appetite. Each circle denotes a neural population that sends or receives signals involved in non-homeostatic appetite. Filled circles include genetically defined populations, whereas broken circles indicate projection targets of unknown identity. Solid lines and dashed lines indicate connections that are orexigenic and anorexigenic when artificially activated, respectively. Split terminals indicate activation of target neurons, and bar terminals indicate inhibition of target neurons. Specific marker genes are indicated by color-coded labels adjacent to regions that contain soma of each population. Note that not all connections depicted here have been confirmed to be monosynaptic. The listed genes encode *Vgat*, vesicular GABA transporter; *Vglut2*, vesicular glutamate transporter 2; *Vglut1*, vesicular glutamate transporter 1; *Pomc*, pro-opiomelanocortin; *Dat*, dopamine active transporter; *Pnoc*, prepronociceptin; *Drd1*, dopamine receptor D1; *Sst*, somatostatin; *Prkcd*, protein kinase c delta; *Nos1*, nitric oxide synthase 1; and *Chat*, choline acetyltransferase. Abbreviations: DS, dorsal striatum; NAc, nucleus accumbens; CeA, central amygdaloid nucleus; InsCtx, insular cortex; BNST, bed nucleus of the stria terminalis; TN, tuberal nucleus of the hypothalamus; PVH, paraventricular hypothalamic nucleus; PVT, paraventricular thalamic nucleus; vBF, ventral subdivision of the basal forebrain; DBB, diagonal band of Broca; LH, lateral hypothalamic area; VTA, ventral tegmental area; peri-LC, peri-locus coeruleus; vSub, ventral subiculum; SNc, substantia nigra pars compacta; PB, parabrachial nucleus; NTS, nucleus of the solitary tract; NG, nodose ganglion.

Mounting evidence suggests that inhibitory neurons in the LH expressing vesicular GABA transporter (encoded by the *Vgat* gene) (LH^*Vgat*^ neurons) comprise a key circuit node for non-homeostatic appetite (Fig. 2). Optogenetic and chemogenetic activation of these neurons increases food consumption and the time spent in a food area, while acute inhibition of this population decreases food intake and the time spent in a food zone^68^. Moreover, ablation of LH^*Vgat*^ neurons reduces body weight^68^. However, in stark contrast to ARC^*Agrp*^ neurons that are implicated in homeostatic appetite, these neurons, at least as a population, appear to not respond to energy deficit nor to serve in restoring caloric deficiency. Fos expression in these neurons was not increased after fasting^69^, implying that this population is not activated upon energy deficit. Activating the LH^*Vgat*^ population elicits consummatory behavior that is not specific to food but also directed toward a wide range of objects, including alcohol, nonnutritive sweeteners, water, and even a wooden stick^70–72^, which may not effectively resolve nutrient deficits. Instead, this population is crucial for non-homeostatic appetite driven by environmental cues^69^. Following chemogenetic activation of these neurons in a specific context, if mice were given chow food in the same context, sated mice consumed more food in the stimulation-paired context in the absence of chemogenetic activation^69^. Interestingly, this ‘contextual feeding conditioning’ driven by LH^*Vgat*^ neuron activation was not established when these neurons were activated without food or with a wooden stick^69^. In line with their role in contextual feeding conditioning, LH^*Vgat*^ neurons transmit positive valence signals, and stimulation of this population is reinforcing^68^. Although LH^*Vgat*^ neurons are sufficient to produce contextual feeding conditioning, inhibition of this population did not impair contextual feeding conditioning driven by high-fat food, wherein a standard chow diet and chemogenetic activation in sated mice during the conditioning phase was replaced with a high-fat diet but no chemogenetic stimulation in fasted mice (in contrast, inhibition of TN^*Sst*^ neurons did impair this conditioning; see below)^69^. Notably, activation of ARC^*Agrp*^ neurons did not yield contextual feeding conditioning^69^.

Multiple projection arms of LH^*Vgat*^ neurons extending to the PVH, diagonal band of Broca (DBB) of the basal forebrain, ventral tegmental area (VTA), and peri-locus coeruleus (peri-LC) are all capable of driving feeding behavior^73–77^. Among these, fibers projecting to the VTA, a midbrain dopamine center critical to reward processing^2,6^, represent a crucial connection through which LH^*Vgat*^ neurons engage the mesolimbic dopamine system to support feeding behavior^73,76^. Activating projections from LH^*Vgat*^ neurons in the VTA increases feeding accompanied by maladaptive gnawing behavior; this aberrant behavior was largely absent during bulk stimulation of LH to VTA projections, which suggests that LH^*Vgat*^ neurons act in coordination with excitatory signals across the LH-VTA pathway to promote appropriate feeding behavior^73^. Bulk stimulation of LH neuronal projections terminating in the VTA increased food consumption even when mice were required to cross an electric shock grid to obtain the food reward, indicating that these projections potently drive compulsive feeding behavior that persists even in the face of punishment^73^. Notably, activation of LH^*Vgat*^ neurons increases dopamine levels in the nucleus accumbens (NAc)^76^, a forebrain region densely innervated by the VTA that shows increased dopamine activity during food-seeking behavior^6,78^. Activation of LH^*Vgat*^ axons innervating the VTA inhibits GABAergic neurons in the VTA, which then disinhibits dopaminergic VTA neurons projecting to the NAc, thereby increasing dopamine levels in the NAc^76^. This increase in dopamine levels in the NAc may play a key modulatory role in promoting the intake of palatable foods^9^.

Another significant population downstream of LH^*Vgat*^ neurons is *Vgat*-expressing neurons in the peri-locus coeruleus (peri-LC^*Vgat*^ neurons); these neurons medially appose the locus coeruleus (LC). Activation of the projections from LH^*Vgat*^ neurons to the peri-LC, as well as activation of peri-LC^*Vgat*^ neurons, evokes voracious feeding behavior^77^. Remarkably, lesions of peri-LC^*Vgat*^ neurons completely block feeding driven by the activation of axons projecting from LH^*Vgat*^ neurons to the peri-LC, indicating that the peri-LC neurons that LH^*Vgat*^ neurons recruit to promote feeding are GABAergic. In contrast, VTA GABAergic neuron lesions did not alter feeding stimulated by activation of LH^*Vgat*^ projections to the VTA^77^. Considering that axons from LH^*Vgat*^ neurons to the peri-LC bypass the VTA, it was suggested that feeding evoked by LH^*Vgat*^-VTA projection stimulation is attributable to activation of LH^*Vgat*^ axon fibers that pass the VTA and terminate in the peri-LC^77^.

Activation of LH^*Vgat*^-DBB projections also induces feeding. In particular, when mice are provided with both normal and high-fat chow, optogenetic stimulation of the projections evokes indiscriminate feeding toward both normal and high-fat diets^74^. In the DBB, the majority of projection target neurons innervated by LH neurons express the cholinergic marker gene encoding choline acetyltransferase (*Chat*)^74^. In line with the notion that LH^*Vgat*^ neurons inhibit DBB^*Chat*^ neurons to drive feeding, optogenetic activation of DBB^*Chat*^ neurons reduces food intake, whereas ablation of these neurons or conditional *Chat* knockout in the DBB increases feeding and body weight^79^. Intriguingly, optogenetic activation of the projections from DBB^*Chat*^ neurons to the ARC reduces feeding, and ablation of cholinergic neurons in the DBB lowers *Pomc* gene expression in ARC^*Pomc*^ neurons^79^. As ARC^*Pomc*^ neurons are activated by acetylcholine, it seems plausible that LH^*Vgat*^ neurons inhibit ARC^*Pomc*^ neurons through DBB^*Chat*^ neurons to promote overconsumption^79^.

In addition to LH^*Vgat*^ neurons and their downstream pathways, several populations in the forebrain and hindbrain regions have also been implicated in non-homeostatic appetite, as activation of these populations selectively increases the intake of palatable foods without affecting that of ordinary food or drives feeding even in a sated state. For example, the projection from the anterior PVT (aPVT) to the NAc preferentially drives the intake of palatable high-fat food over standard chow food^80^. Activation or inhibition of aPVT-NAc projections promotes or suppresses consumption of a high-fat diet, respectively, without affecting standard chow feeding^80^. Within the ventral subdivision of the basal forebrain (vBF), defined as the region including the DBB, the substantia innominata, and magnocellular preoptic nucleus, *Sst*-expressing neurons (vBF^*Sst*^ neurons) promote the intake of palatable food^81^. Activating these neurons in mice specifically increases high-fat chow intake and sucrose preference without affecting the consumption of standard chow^81^. Interestingly, activating vBF^*Sst*^ neuronal projections to the LH significantly increases the intake of high-fat chow but does not affect sucrose preference, revealing a putative dissociation of preference for fat and sugar within the category of palatable food^81^.

Some hypothalamic neurons within and proximal to the ARC also drive palatable feeding. Inhibitory neurons in the ARC expressing the *Pnoc* gene (which encodes prepronociceptin) (ARC^*Pnoc*^ neurons) are also important in driving palatable food intake^82^. These neurons are distinct from ARC^*Agrp*^, ARC^*Th*^, and ARC^*Pomc*^ neurons, and these neurons are activated upon high-fat food intake but not upon standard chow intake^82^. Activation of these neurons or their projections to the BNST increased standard chow intake in animals maintained on a standard chow diet, and stimulating these neurons in high-fat-fed animals increased consumption of a high-fat diet. In addition, activating these neurons elicited direct GABAergic inhibition of neighboring ARC^*Pomc*^ neurons^82^. Conversely, selective ablation of these cells reduces high-fat feeding and body weight gain in mice maintained on a high-fat diet for three days, but it does not affect food intake or body weight under standard chow consumption^82^. On the other hand, activation of TN^*Sst*^ neurons in sated mice and inhibition of these neurons in hungry mice increased and decreased the intake of palatable food, respectively, without affecting standard chow consumption^69^. Chemogenetic activation of these neurons elicits a preference for the stimulation-paired chamber^50^.

Recently, neurons expressing vesicular glutamate transporter 2 (*Vglut2*) in the hindbrain peri-LC (peri-LC^*Vglut2*^ neurons) have been implicated in palatability-driven feeding^83^ (the authors of this work have defined the peri-LC as the pontine region medial to the anterior locus coeruleus, lateral to the laterodorsal tegmental area, and ventral to the fourth ventricle^83^; this definition largely overlaps with the medial ‘peri-LC’ densely populated by peri-LC^*Vgat*^ neurons noted above but also includes regions dorsally apposing the LC^77,83^). Recording of peri-LC^*Vglut2*^ neurons in vivo revealed that the majority of these neurons are inhibited during food consumption, and this inhibition was stronger during the consumption of more palatable substances^83^. Inhibition of these neurons promotes feeding and conditions place preference^83^. Interestingly, optogenetic inhibition of peri-LC^*Vglut2*^ neurons increases food intake by sustaining food consumption only when light delivery is temporally coincident with spontaneously initiated bouts, rather than by triggering a bout. It was concluded that this circuit node supports a palatability-guided double-negative feedback model, where consumption of palatable substances inhibits these neurons, which in turn serves to prolong consummatory action^83^.

Notably, palatability-driven feeding can occur in the absence of ARC^*Agrp*^ neurons^28,29^. Selective ablation of ARC^*Agrp*^ neurons dramatically impaired standard chow intake but spared the consumption of several different kinds of palatable diets^28,29^. In line with this, body weight loss observed in mice lacking ARC^*Agrp*^ neurons is prevented when these mice are fed palatable food^28^.

As illustrated above, many neuronal populations promote feeding specific to palatable food. However, it is noteworthy that food with palatable flavor, in most cases, is also dense in nutrients. Indeed, animals can acquire preferences for specific flavors that are paired with nutrients^13^. Unsurprisingly, a series of studies have investigated neural circuit mechanisms by which nutrient signals from the gut engage the central dopaminergic system to establish preferences for nutrient-rich food and its associated cues, which may later trigger non-homeostatic appetite^12,13^.

In the ventral striatum, dopamine levels rise upon sucralose (a nonnutritive sweetener) licking alone but not upon licking of sucralose mixed with bitter tastants^84^. In contrast, dopamine levels in the dorsal striatum are not increased by sucralose licking per se but are increased when sucralose licking is paired with nutrient infusion into the gut^84^. When sucralose licking is associated with noncaloric sugar, the dorsal striatal dopamine level is not altered^84^. These results indicate that the ventral striatal dopamine level relates to oral sweetness, while dorsal striatal dopamine release represents gut nutrients. Moreover, ablation of dopamine D1 receptor-expressing dorsal striatum neurons (DS^*D1r*^ neurons) revealed that these neurons are required for the preference of nutrient-paired flavor^84^. When bitter-sweet mixture solution was paired with intragastric infusion of nutrients and sweet solution was paired with the infusion of sucralose, mice with ablated DS^*D1r*^ neurons preferred sweet solution over bitter-sweet mixture, whereas neither wild-type mice nor mice with ablated dopamine D1 receptor-expressing ventral striatum neurons (VS^*D1r*^ neurons) preferred sweet solution^84^. Conversely, optogenetic activation of DS^*D1r*^ neurons, but not VS^*D1r*^ neurons, robustly increased the intake of both sweet and bitter solutions, as well as another nonnutritive sweetener that was previously associated with visceral malaise^84^. Taken together, these results indicate that dopamine in the dorsal striatum is a key signal that underlies flavor-nutrient conditioning.

Furthermore, a remarkable recent study proposed the pathway from the gut to the dorsal striatum that signals the presence of fat in the gut^85^. Anterograde and retrograde transsynaptic tracing experiments have revealed an anatomical polysynaptic pathway originating from the gut and spanning across the right vagus nerve to the NTS, PB, substantia nigra pars compacta (SNc), and dorsal striatum^85^. Interestingly, between the right and left vagus nerve, the right one appears to carry fat signals that have positive valence and are reinforcing; the stimulation of the right, but not the left, nodose ganglion neurons that project to the NTS elicited a preference for place paired or conditioned with stimulation, sustained self-stimulation behavior and induced dopamine release upon intragastric fat infusion in the dorsal striatum^85^. Consistent with the anatomical tracing results, activating SNc-projecting PB neurons also recapitulated these effects^85^. Ablation of either ascending fibers from the right nodose ganglion or SNc-projecting PB neurons impaired the preference for flavor paired with intragastric fat infusion and abolished the increase in dopamine evoked by intragastric fat infusion^85^.

In the case of intragastric infusion of sucrose, VTA dopamine neurons were found to be activated^86^. This increased VTA dopamine neuron activity is impaired by transection of the hepatic branch of the vagus nerve (a branch that diverges from the left vagal trunk), whereas optogenetic stimulation of the neurons in the left nodose ganglion increases VTA dopamine activity^86^.

The presence of food-predictive environmental cues sufficiently triggers feeding behavior in the absence of hunger, and recent studies have identified genetically defined neural populations necessary for overconsumption in a conditioned context. Within the hypothalamus, activation of TN^*Sst*^ neurons as well as that of LH^*Vgat*^ neurons is sufficient for establishing contextual feeding conditioning^69^. However, only activation of TN^*Sst*^ neurons, but not LH^*Vgat*^ neurons, is necessary for context-driven overconsumption of palatable foods^69^. Both neural populations receive monosynaptic inputs from the ventral subiculum (vSub), a hippocampal region shown to transmit contextual information^69^. Interestingly, the amplitude of postsynaptic currents in TN^*Sst*^ neurons evoked by activation of vSub-TN axons was elevated in animals subjected to contextual conditioning for palatable food, whereas no conditioning-dependent synaptic plasticity was observed in LH^*Vgat*^ neurons^69^. Inhibition of TN^*Sst*^ neurons, but not ARC^*Agrp*^ neurons or LH^*Vgat*^ neurons, reduces the intake of palatable food in a conditioned context, highlighting the necessity of establishing contextual feeding conditioning driven by palatable food^69^.

Context-dependent overconsumption also relies on a top-down circuit extending from the cerebral cortex. In the insular cortex (InsCtx), neurons expressing nitric oxide synthase 1 (encoded by the *Nos1* gene) (InsCtx^*Nos1*^ neurons) that project to the central amygdala (CeA) are required for overconsumption of chow food in a conditioned context^87^. Curiously, while InsCtx^*Nos1*^ neurons projecting to the CeA are primarily glutamatergic, these neurons provide net inhibition of anorexigenic neurons expressing PKCδ (encoded by the *Prkcd* gene) in the CeA, possibly by recruiting inhibitory interneurons within the CeA^87,88^.

## Interactions between homeostatic and non-homeostatic appetites

The current view on homeostatic and non-homeostatic appetites is that these engage overlapping neural circuits that jointly promote feeding behavior^1,6^. Recent studies have provided unprecedented insight into the circuit mechanisms that underlie the interactions between homeostatic and non-homeostatic appetite regulation^1,6,11^.

It is well known that hunger increases the reward value of food^1,2,4,6,9,11,13^. Consistently, ghrelin and leptin have been found to modulate dopamine reward circuits. Infusion (i.c.v.) of ghrelin potentiated food-evoked dopamine release in the NAc^89^, whereas leptin blunted the food cue-evoked activity observed in VTA dopamine neurons in hungry animals^90,91^. Moreover, chemogenetic activation of ARC^*Agrp*^ neurons potentiates dopamine responses upon food presentation in the NAc^92^. Significantly, ARC^*Agrp*^ neuron stimulation without food presentation did not change the baseline dopamine level in the NAc^92^.

Conversely, dopaminergic reward signaling plays a causal role in homeostatic appetite. Systemic injection of a D2R antagonist or genetic knockout of D2R blunts the hypophagic effect of systemic leptin injection^93^. In contrast, how dopamine signaling modulates the orexigenic effect of ghrelin is unclear, as both agonism and antagonism of central D1R or D2R/D3R receptors attenuated the orexigenic effects of i.c.v. ghrelin injections^94^, whereas the hyperphagic effects of peripheral ghrelin administration were not affected by systemic injection of D1R or D2R antagonists^93^. Remarkably, blockade of dopamine receptor signaling by systemic injection of D1R and D2R antagonists attenuates the response of ARC^*Agrp*^ neurons to nutrients^92^. Moreover, systemic injection of a dopamine reuptake inhibitor in mice lacking ARC^*Agrp*^ neurons can restore standard chow intake^28^, implying that dopamine signaling can override the absence of ARC^*Agrp*^ neurons and promote standard chow intake.

As in the case of TN^*Sst*^ neurons, a single population can also be involved in homeostatic and non-homeostatic control of appetite^50,69^. These neurons are activated in the hunger state, as well as by ghrelin signaling an energy deficit, and activation of these neurons promotes chow food intake^50^. At the same time, these neurons are necessary for food consumption beyond the energy need that occurs in the context paired with high-fat food^69^. This notable case exemplifies that neural circuits for homeostatic and non-homeostatic appetites are not entirely dissociable.

## Conclusion and future directions

Here, we reviewed studies on the neural circuit mechanisms driving homeostatic and non-homeostatic appetites, with an emphasis on recent findings. We also discussed that the neural systems supporting the two modes of appetite interact with each other and share common circuit nodes. A key objective in the study of neural circuit mechanisms underlying appetite regulation is identifying specific roles played by a particular neural population. Diverse experimental paradigms have illuminated how individual neural populations mediate particular aspects of feeding behavior. Employing the relevant methodologies to characterize neural populations affecting feeding behaviors would aid in the comprehensive elucidation of their functional significance. Among the numerous approaches, we summarize some notable examples and the possible interpretations that can result:

- Recording the activity of neurons during energy deficit and surfeit, or in response to the changes in humoral signals such as hormones or nutrients, would reveal their possible role in sensing physiological energy states.
- Assessing the effects of stimulating a neural population on the intake of diets of different nutritional compositions (e.g., high-fat, high-sugar, and high-protein diets) may uncover their causal role in nutrient-specific appetites.
- Investigating the activity of a neural population during ingestion of nonpalatable and palatable foods will help determine its role in palatability-driven feeding.
- Testing if these neurons are activated by food-predictive cues, if these cues are sufficient or necessary for the learning of food-cue associations, and if this neural population promotes overconsumption in the presence of learned cues can verify their involvement in cue-evoked conditioned overconsumption.
- Detailed analysis of meal structure (e.g., bout number, bout duration, latency to the first bout) following perturbation of a neural population can help pinpoint where its orexigenic or anorexigenic effect is exerted along the sequence of feeding behavior (i.e., initiation and termination) and aid in the identification of their specific role in regulating ingestive behaviors (i.e., increase motivation to initiate feeding, decrease motivation to stop feeding).
- Observing the long-term (i.e., days to weeks) effects of ablating or stimulating specific neurons would be informative in determining their necessity or sufficiency in promoting feeding behavior in the long term, which may bear higher clinical relevance.

In addition, multiple neuropeptide systems (e.g., the melanocortin, neuropeptide Y, and opioid systems) are heavily involved in the control of appetite and should thus be taken into account in this research. Beyond studying each circuit one at a time, elucidating the cooperative workings of the many circuit components in a network context remains a salient future task for revealing a diagram of the full neurocircuitry that drives food intake. Cutting-edge techniques enabling interrogation of neural circuit elements in intact brain tissues would further accelerate this research endeavor^14–16,95–97^.

From a clinical perspective, it would be critical to examine how neural activity, synaptic connectivity, or transcriptomic profiles of each orexigenic population are affected in disease conditions such as obesity or anorexia nervosa. It is equally important to investigate how pharmacological (e.g., GLP1R agonists) or surgical interventions (e.g., gastric bypass surgery) for such diseases affect appetite-promoting circuit nodes, which would lead to crucial translational insights^3,98–101^. Furthermore, identification of the neural circuits responsible for such disorders and subsequent screening of druggable targets specific to these neurons by means of single-cell RNA sequencing holds great promise in opening novel avenues for the treatment of feeding pathologies^3,4,15,102–105^.
